# Next-Generation cDNA Screening for Oncogene and Resistance Phenotypes

**DOI:** 10.1371/journal.pone.0049201

**Published:** 2012-11-07

**Authors:** Nobuaki Shindoh, Akinori Yoda, Yuka Yoda, Timothy J. Sullivan, Oliver Weigert, Andrew A. Lane, Nadja Kopp, Liat Bird, Scott J. Rodig, Edward A. Fox, David M. Weinstock

**Affiliations:** 1 Department of Medical Oncology, Dana-Farber Cancer Institute, Boston, Massachusetts, United States of America; 2 Microarray Core, Dana-Farber Cancer Institute, Boston, Massachusetts, United States of America; 3 Harvard Medical School, Boston, Massachusetts, United States of America; 4 Department of Pathology, Brigham and Women's Hospital, Boston, Massachusetts, United States of America; 5 Drug Discovery Research, Astellas Pharma Inc., Tsukuba, Ibaraki, Japan; Virginia Commonwealth University, United States of America

## Abstract

There is a pressing need for methods to define the functional relevance of genetic alterations identified by next-generation sequencing of cancer specimens. We developed new approaches to efficiently construct full-length cDNA libraries from small amounts of total RNA, screen for transforming and resistance phenotypes, and deconvolute by next-generation sequencing. Using this platform, we screened a panel of cDNA libraries from primary specimens and cell lines in cytokine-dependent murine Ba/F3 cells. We demonstrate that cDNA library-based screening can efficiently identify DNA and RNA alterations that confer either cytokine-independent proliferation or resistance to targeted inhibitors, including RNA alterations and intergenic fusions. Using barcoded next-generation sequencing, we simultaneously deconvoluted cytokine-independent clones recovered after transduction of 21 cDNA libraries. This approach identified multiple gain-of-function alleles, including KRAS G12D, NRAS Q61K and an activating splice variant of ERBB2. This approach has broad applicability for identifying transcripts that confer proliferation, resistance and other phenotypes *in vitro* and potentially *in vivo*.

## Introduction

Thousands of somatic variants have recently been catalogued across multiple cancer types. However, the functional relevance of nearly all of these variant alleles remains undefined and only a small minority are likely to contribute to malignant phenotypes. Thus, approaches are broadly needed to distinguish which of the genetic variants present in both coding and noncoding RNA have functional relevance, and may therefore represent therapeutic targets [1].

Inhibition of oncogene function can induce cell death and tumor regression across a variety of cancers but is almost invariably associated with the selection of resistant clones. Multiple genetic and epigenetic mechanisms can mediate resistance to targeted inhibitors, including mutation of the therapeutic target, activation of downstream signaling pathways and upregulation of orthogonal pathways. Similar to the identification of oncogenes, delineating the multiple possible mechanisms of resistance to targeted inhibitors can be labor intensive and has primarily involved biased approaches that only interrogate protein-coding genes [2].

The development of methods for cloning full-length cDNA [3] and constructing cDNA libraries [4] in the early 1980s led to a series of phenotype-based screens that broadly interrogated the transcriptome. For example, the Rennick laboratory identified a cDNA for human GM-CSF by transfecting cDNA libraries derived from activated human T-cells into COS-7 monkey cells [5]. The Nolan laboratory used Ba/F3 cells to isolate clones that expressed selectable markers like hCD2 or hIL3R after retroviral transduction of cDNA libraries [6]. Ba/F3 cells are murine hematopoietic cells that are absolutely dependent on IL3 for survival. Expression of some oncogene transcripts can substitute for IL3 signaling, allowing for the selection of monoclonal populations that proliferate in the absence of IL3.

cDNA libraries have also been used to identify new oncogenes from tumor cell lines and primary samples. In 1994, Chan et al. identified a novel RAS superfamily member by screening an ovarian cancer cell line cDNA library in 3T3 cells. Nearly fifteen years later, the Mano laboratory used essentially the same protocol to identify the EML4-ALK fusion from a lung cancer sample [7]. We previously discovered a mutant allele of the CRLF2 cytokine receptor by screening cDNA libraries generated from primary acute lymphoblastic leukemias (ALL), which multiple groups concurrently identified as an important oncogene in poor-risk ALL [8], [9].

Screens of cDNA can identify alterations present within exonic DNA as well as splice alterations, RNA editing events and noncoding RNAs. However, standard approaches for generating and screening high-quality cDNA libraries have multiple shortcomings. First, up to 5 µg of mRNA may be required, which precludes the use of most primary tumor specimens. Second, first-strand synthesis may prematurely abort and thereby generate truncated cDNA that are not present within the sample. Third, the isolation and analysis of individual clones with the desired phenotype can be very cumbersome. Finally, target cells that have a background rate of spontaneous transformation (e.g. 3T3) can generate multiple false positive clones, further reducing the efficiency of cDNA library screening.

We developed new methods for efficiently generating high-quality libraries of full-length cDNA using ≤1 µg total RNA. We screened a panel of primary specimen and cell line libraries in Ba/F3 cells to identify transcripts with either DNA or RNA alterations that could substitute for IL3 signaling. To deconvolute the integrated cDNA from pools of clones, we developed methods for next-generation sequencing and bioinformatic analysis that greatly improve the efficiency of cDNA library-based screening. These approaches can be applied to screen both primary and immortalized samples for a variety of phenotypes.

## Results and Discussion

To generate high-quality, full-length cDNA libraries, we created a ‘Full length-att’ protocol that hybridizes the In-Fusion SMARTer Directional cDNA Library Construction Kit (Clontech) with recombinase reactions (Invitrogen) for shuttling between vectors (Figure S1). We perform quality control of these libraries in three steps. First, libraries are transformed into *E. coli* to confirm the presence of ≥10^7^ clones per library. Second, restriction enzyme digestion of each library is performed to demonstrate clonal heterogeneity (Figure S1). Third, we developed quantitative PCR (qPCR) approaches using primers that amplify the 5′ region of housekeeping genes to confirm that similar frequencies of long (e.g. REV3L, 10719 bp) and short (e.g. GAPDH: 1401 bp) full-length cDNAs are present (Figure S1). We generate libraries in medium-throughput fashion (*i.e.*, batches of 12) and screen for IL3-independent growth and drug resistance phenotypes in Ba/F3 cells, as previously performed [10].

**Figure 1:**
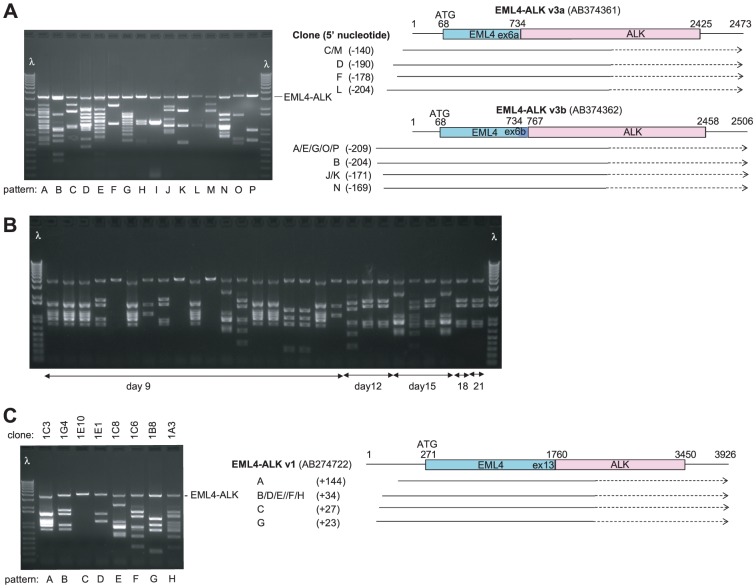
cDNA library screening identifies EML4-ALK. **A.** Screening of a library generated from H2228 cells generated 16 clones with distinct patterns of integrated cDNA. All clones contained one of two EML4-ALK transcripts that differed based on splicing of exon 6 and the length of the 5′ untranslated region, as indicated by the most 5′ nucleotide fused to the attB sequence. λ indicates size ladder. **B.** Screening of a library generated from H3122 cells generated Ba/F3 clones isolated at the indicated time point after withdrawal of IL3. PCR is shown using primers that amplify integrated cDNA. **C.** PCR amplification from eight clones generated from the H3122 library with different patterns of integrated cDNA. Five distinct EML4-ALK transcripts were identified based on differences in the length of the 5′ untranslated region, as in **A.**

To validate this approach, we screened cDNA libraries generated from the lung cancer cell lines H3122 and H2228 (Figure 1), which both harbor EML4-ALK fusions. All IL3-independent Ba/F3 clones recovered after transduction of both libraries harbored full-length EML4-ALK and were sensitive to the ALK inhibitor crizotinib (LD_99_<1 µM). Multiple independent EML4-ALK transcripts were recovered from both libraries, based on the presence of different lengths of 5′ untranslated region (UTR), which results from variable elongation past the translation start site during first-strand cDNA synthesis (Figure 1A). Two distinct EML4-ALK fusion transcripts were identified from H2228, which differed based on alternate splicing of exon 6 (Figure 1A). Thus, this approach can efficiently recover multiple gain-of-function transcripts from the same tumor specimen.

**Figure 2:**
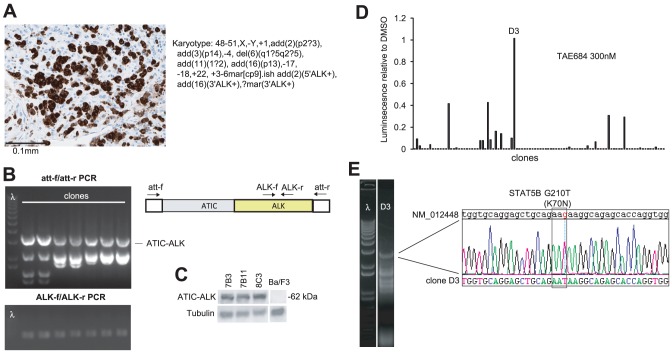
cDNA library screening identified an ALK translocation partner and resistance allele. **A.** Immunohistochemistry using an antibody against ALK and karyotype of the anaplastic large cell lymphoma specimen 421. **B.** Multiple IL3-independent clones were recovered from screening of the cDNA library generated from 421, all of which contained ALK cDNA using primers within the flanking attB sites or within ALK itself. **C.** Immunoblot using antibody against ALK demonstrates ATIC-ALK expression in multiple clones but not in wild-type Ba/F3 cells. **D.** Effect on proliferation of 96 individual clones recovered from the 421 library with TAE684 300 nM, quantified using the ATP chemiluminescent agent CellTiterGlo. **E.** PCR amplification using the att-f and att-r primers with DNA from clone D3 demonstrates a full-length STAT5B allele that harbored a G210T (K70N) mutation.

To identify an unknown translocation fusion partner, we screened a cDNA library isolated from a biopsy of a primary anaplastic large cell lymphoma (ALCL) that expressed the ALK oncoprotein but did not have the common NPM-ALK rearrangement (Figure 2). We recovered multiple Ba/F3 clones that all contained and expressed a full-length ATIC-ALK fusion transcript (Figure 2B–C), which was previously described in a small subset of ALCLs [11]. We isolated 96 IL3-independent Ba/F3 clones after transduction of this library and tested the effects of the specific ALK inhibitor TAE684 on proliferation. Of the 96 clones, only clone D3 was unaffected by 1 µM TAE684 (Figure 2D). Sequencing of the integrated cDNA within D3 identified a full-length STAT5B transcript that harbored a K70N mutation in the N-terminal region (Figure 2E). Thus, clone D3 had acquired ALK-independent proliferation in the absence of IL3.

**Figure 3:**
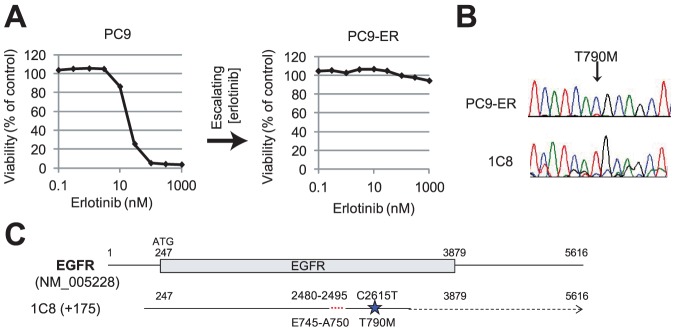
Screening for resistance alleles. **A.** Selection of PC9 cells in media containing increasing concentrations of erlotinib resulted in the PC9-ER line, which is unaffected by doses of erlotinib up to 1 µM. **B.** The IL3-independent Ba/F3 clone 1C8 recovered by cDNA library screening of PC9-ER cells contains both the Δex19 activating mutation and the T790M mutation in EGFR. **C.** Sanger sequencing shows that only a small fraction of EGFR amplified from PC9-ER cells harbors the T790M alteration, in contrast with 1C8.

We also utilized cDNA library screening to recover alleles that confer resistance to targeted inhibitors. First, we generated the PC9-ER cell line by culturing PC9 lung cancer cells, which express the transforming EGFR delE745-A750 allele, in the presence of escalating doses of the EGFR inhibitor erlotinib (Figure 3A). As previously described, this resulted in subclonal outgrowth of PC9 cells harboring the gatekeeper mutation T790M, which can be difficult to discern by Sanger sequencing (Figure 3B) [12]. Screening of a cDNA library generated from PC9-ER in Ba/F3 cells resulted in multiple full-length clones that harbored EGFR with both delE745-A750 and T790M (Figure 3C) and were sensitive to the mutant-selective EGFR inhibitor WZ4002 (LD_99_<1 µM) [13].

Transduction of Ba/F3 cells at a high multiplicity of infection (MOI) allows for screening of >10^7^ transcripts in medium-throughput fashion, but the IL3-independent Ba/F3 clones typically harbor 5–15 distinct cDNA inserts (Figure 1). To functionally deconvolute inserts from IL3-independent Ba/F3 clones, we developed methods for insert identification by next-generation sequencing. To test this strategy, we generated 21 cDNA libraries from primary specimens and cell lines and transduced each library into 5×10^6^ Ba/F3 cells. After withdrawal of IL3, proliferating clones were subjected to PCR amplification of integrated cDNA, followed by barcoding by library, pooling and sequencing on the SOLiD 4 platform. Approximately 18 Gbp of sequence data was mapped to the human reference genome (hg18). As genes with functional relevance are typically present in multiple clones with different 5′ UTRs (Figure 1), we developed a bioinformatic pipeline for the detection of attB fusion sequences and precisely mapped the genomic coordinates of the integrated cDNA.

**Figure 4:**
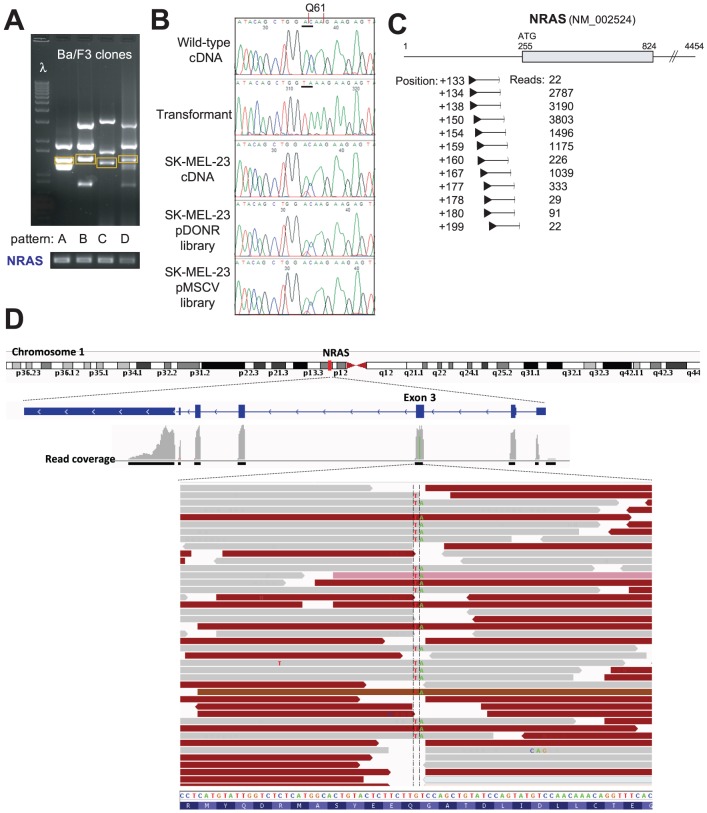
Deep sequencing deconvolution of multiple cDNA inserts. **A.** Screening of the SK-MEL-23 melanoma cell line results in multiple clones with different patterns of cDNA inserts (upper) that all harbor NRAS based on an NRAS specific PCR (lower). **B.** The mutant NRAS allele harbors an A434T (G60G) and C435A (Q61K) dinucleotide substitution (underlined by thick black bar) that is present as 100% of sequence within a BaF3 transformant and as 50% or less in the SK-MEL-23 cDNA, plasmid acceptor library and retroviral library. **C.** Deep sequencing identified 12 distinct fusion sequences between the attB (black triangle) and 5′ UTR of NRAS from the SK-MEL-23 library. The number of individual reads for each of the sequences is provided. **D.** A subset of approximately 90,000 reads across the NRAS dinucleotide substitution are shown. The sequence is on the genomic negative strand and thus reversed from **C.** The C435A (G→T shown on the positive strand) substitution that results in Q61K is between the hashed marks. Gray reads have the paired-end on the same exon while brown reads have the paired-end on the next exon.

Among the 21 libraries were two positive controls, which were known to harbor activating RAS family alleles. Library transduction followed by PCR amplification of integrated cDNA from IL3-independent clones and next-generation sequencing successfully identified both KRAS G12V from the small cell lung carcinoma line SHP-77 and NRAS Q61K from the melanoma cell line SK-MEL-23 (Figure 4). From the latter, 12 independent fusions of attB sequence with the 5′ UTR of NRAS, representing full-length transcripts, were recovered in amplified fragments (Figure 4C).

NRAS was also the most abundant transcript in amplified fragments from the SK-MEL-23 library, with a fragments per kilobase of exon per million fragments mapped (FPKM) value of 61516. The next 7 most abundantly recovered genes (PKM2, TKT, ACTG1, RPL11, AP4M1, OAZ2, IMPDH2) are all housekeeping genes and were present at FPKM values between 10165–14711. From amplified fragments recovered from the SHP-77 library, KRAS was the 5^th^ most abundant transcript (FPKM 30645), behind the non-coding transcript GAS5 (FPKM 56640) and the genes UBE2V2, CHMP5, RPL18 and RPL19 (FPKM 36338–48842). No variants were present in the coding sequence of any of these 4 genes. Thus, the identification of mutated transcripts expressed at high FPKM can highlight driver alterations with this method.

**Figure 5:**
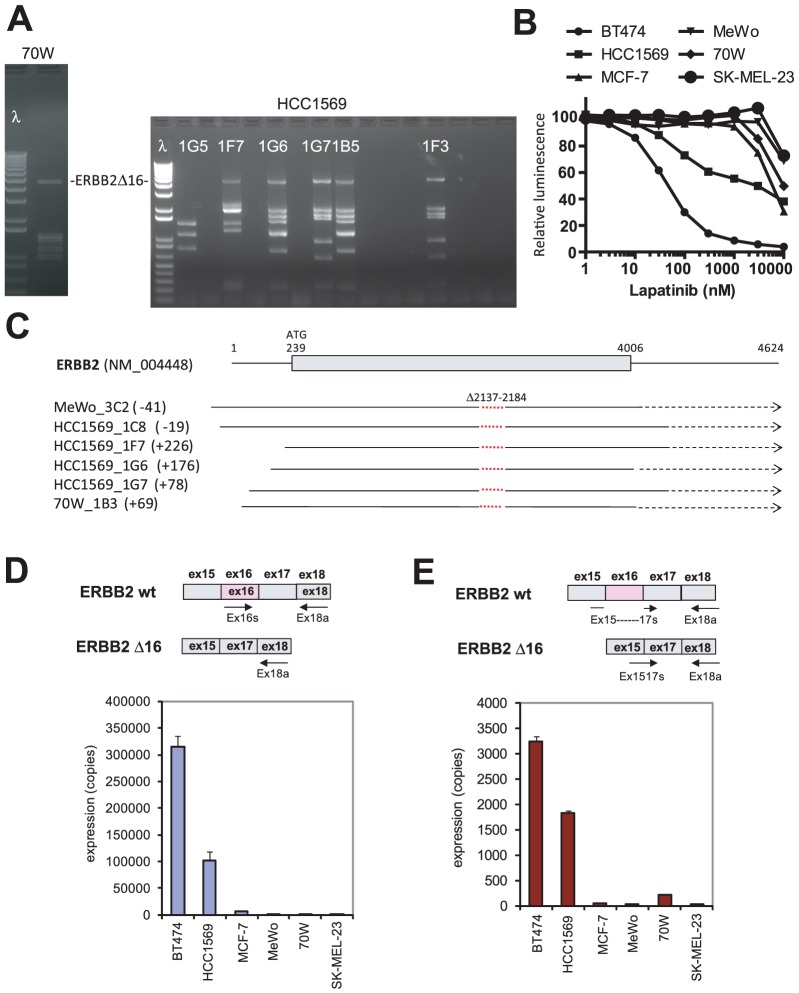
Recovery of a transforming ERRB2 splice variant. **A.** cDNA library screening of the melanoma cell lines 70 W (a sub-line of the MeWo) and HCC1569 resulted in IL3 independent Ba/F3 clones that contained ERBB2Δ16. **B.** Relative proliferation quantified using the ATP chemiluminescent agent CellTiterGlo. **C.** cDNA transcripts recovered from screening of MeWo, HCC1569 and 70 W cell lines. All of the transcripts lacked amino acids 2137–2184 that comprise exon 6. **D.** Expression of ERBB2 cDNA containing exon 16 using specific primers. Error bars indicate standard deviation. **E.** Expression of ERBB2Δ16 cDNA using specific primers, including a 5′ primer that overlaps exons 15 and 17 so does not amplify wild-type ERBB2.

Next-generation sequencing-based deconvolution identified a splice variant of ERBB2 that lacks exon 16 (ERBB2Δ16) from the breast carcinoma line HCC1569 (Figure 5). Expression of ERBB2Δ16 results in resistance to the ERBB2-specific monoclonal antibody trastuzumab and constitutive activation of SRC [14]. ERBB2Δ16 was also recovered from the melanoma cell lines MeWo and 70 W (Figure 5C). Transcripts isolated from each line had different 5′ UTR sequences, indicating full-length transcripts and confirming that they derived from independent lines (Figure 5C). Although ERBB2Δ16 was detectable in MeWo and 70 W cells, it was expressed at levels 10–1000-fold lower than in the trastuzumab-resistant breast cancer cell lines HCC1569 and BT474 [14] by qPCR (Figure 5D, 5E). ERBB2Δ16 was not among the top 50 transcripts recovered by next-generation sequencing (based on FPKM). Finally, neither melanoma MeWo nor 70 W was sensitive to the EGFR/ERBB2 inhibitor lapatinib (Figure 5B). This suggests that ERBB2Δ16 is not a primary driver of proliferation in MeWo and 70 W cells but could potentially compensate upon targeted inhibition of other pathways.

In conclusion, cDNA library screening with deconvolution by next-generation sequencing can efficiently identify fusions, intragenic alterations and splice variants from primary specimens and cell lines in medium throughput. These approaches are amenable to screens both *in vitro* and potentially *in vivo*, either to agnostically interrogate cDNA libraries or to identify individual transcripts within selected pools for proliferation, resistance and other phenotypes.

## Methods

### Ethics statement

All specimens were acquired from patients at the Dana-Farber Cancer Institute. This study was approved by the Dana-Farber Cancer Institute Institutional Review Board (DFCI IRB #01-026). No research was conducted outside of the United States. All participants provided written consent under the aforementioned protocol.

### Cell lines and media

Ba/F3 cells were obtained from RIKEN. SHP-77, HCC1569 and BT-474 were obtained from ATCC. H3122, H2228 and PC9 were previously reported [15]. MeWo, 70 W and SK-MEL-23 were previously reported [16], [17] and were a kind gift of Dr. David Fisher (Massachusetts General Hospital). All cells were cultured according to published conditions. Immunohistochemistry for ALK was performed as previously described [18].

### RNA isolation and full-length-att cDNA library construction

For construction of full-length cDNA libraries, total RNA was purified using TRIzol reagent (invitrogen) or the RNeasy Mini Kit (Qiagen) followed by DNase I treatment according to the manufacturer's instructions. ≤1 ug total RNA was used for cDNA library construction using a “Full length-att” approach that hybridizes the In-Fusion SMARTer Directional cDNA Library Construction Kit (Clontech) with recombinase reactions (Invitrogen) for shuttling between vectors. The In-Fusion SMARTer Directional cDNA Library methodology was performed with the following modifications. PCR primers were designed to incorporate attB1 recombinase recognition sites during amplification of the SMARTer first-strand cDNA:

attB1_forward: 5′-GGGGACAACTTTGTACAAAAAAGTTGGAAGCAGTGGTATCAACGCAGAG-3′

attB2_reverse: 5′-CCGCACAACTTTGTACAAGAAAGTTGGGTCGGGGTACGATGAGACACCA-3′

Long distance-PCR was carried out with PrimeSTAR HS DNA polymerase (Takara) using the following PCR conditions: 95°C for 1 min, 15 cycles of 98°C for 10 sec, 55°C for 5 sec and 68°C for 10 min. After purification according to the manufacturer's instructions, 300 ng of ds cDNA was cloned into 300 ng of pDONR222 vector with Gateway BP Clonase II enzyme mix (Invitrogen). The retroviral Gateway destination vector pMSCVpuroATT was constructed by inserting Reading Frame Cassette A into the retroviral expression vector pMSCVpuro (Clontech) using the Gateway Vector Conversion System (Invitrogen). Libraries were transferred from the pDONR222 backbone to pMSCVpuroATT using Gateway LR Clonase (Invitrogen). After transformation into ElectroMAX DH10BT1 Phage Resistant *E. coli* (Invitrogen), each pMSCVpuroATT library was cultured at 30°C for 16 hour and purified with thePureLink HQ Midi Plasmid Purification Kit (invitrogen), as described [10].

For libraries constructed with the CloneMiner II cDNA construction kit (Invitrogen), high-quality mRNA was isolated from total RNA with the FastTrack2.0 mRNA Isolation Kit (Invitrogen) according to the manufacturer's instructions. Up to 5 µg mRNA was used according to the manufacturer's instructions. Recombination of attB-flanked cDNA was performed into the attP-containing pDONR222 vector to create a high titer entry library. This library was then transferred into pMSCVpuroATT, as above.

### Library quality control

All libraries were confirmed to contain ≥10^7^ clones by transformation into E.coli and heterogeneity was confirmed by BsrGI digest (Figure S1). Adequate representation of long full-length cDNA was confirmed within cDNA libraries using qPCR with primers that amplified the 5′ region of long (TFRC: 5241 bp, POLQ: 8787 bp, REV3L: 10719 bp) and short (GAPDH: 1401 bp, ACTB: 1852 bp) housekeeping genes.

GAPDH-F: 5′-GTCAGCCGCATCTTCTTTTG-3′; 5′-ACGACCAAATCCGTTGACTC-3′

ACTB: 5′-GATGCAGAAGGAGATCACTGC-3′; 5′-TGATCCACATCTGCTGGAAG-3′

TFRC #1: 5′-AGTGATTGTCAGAGCAGGGAAA-3′; 5′-CCCAGATGAGCATGTCCAAA-3′

TFRC #2: 5′-GAGTGTGAGAGACTGGCAGGAA-3′; 5′-CGGTGAAGTCTGTGCTGTCC-3′

POLQ #1: 5′-GGCAGCACCTCTCCATCAA-3′; 5′-TCATCCACAACCACCATTCC-3′

POLQ #2: 5′-TGTGGCTTCCTGGTTGAATG-3′; 5′-GGGCTCAAATTCCCTCACAA-3′

REV3L #1: 5′-AAGTGGATGCTGTAGCTGCTGA-3′; 5′-GCCGTTGCTTTTCATCTTCC-3′

REV3L #2: 5′-TCTGGCTGCTGTCAAGTTCC-3′; 5′-TCATCTTGTTCCCACCGAAA-3′

### Retroviral cDNA library screening

293T cells (7×10^5^ cells in 2 mL of Dulbecco's Modified Eagle Medium supplemented with 10% fetal calf serum) were inoculated into a single well of a 6-well plate. After 24 hours, 3 µg of pMSCVpuroATT cDNA library and 1 µg of pEcoPack vector were co-transfected into 293T cells with 10 µL of Lipofectamine2000 transfection reagent (invitrogen) and 500 µL of Opti-MEM I Reduced Serum Media (Invitrogen). On the following day, 2.5 mL of fresh medium was added to the cells. After an additional day, the supernatant was passed through a 0.45 µm filter. Before the infection, sub-confluent Ba/F3 cells were diluted 100-fold and then cultured for 3 days in log-growth phase using RPMI-1640 medium supplemented with 10% FCS, 0.5 ng/mL of mouse IL3 (Prospec), penicillin and streptomycin. 2.5 mL of viral supernatant was applied to 5×10^6^ Ba/F3 cells in 2.5 mL, along with 16 µg/mL of polybrene, 10 mM of HEPES-HCL and 0.5 ng/mL of mouse IL3 in a single well of a 6-well plate. The cells were centrifuged at 2500 rpm for 90 min at 37°C. Infected cells were washed once with media containing IL3 on the next day and 1 µg/mL of puromycin was added to the cells 2 days after infection. 48 hours later (4 days after infection), the cells were washed 3 times and 3–4×10^4^ cells were plated into a 96-well plate with puromycin containing media without mouse IL3. After 10–28 days, visible colonies were isolated. Alternatively, cells were washed 96 hours after infection and transferred to a flask containing media without IL3. Proliferating cells were visible 21–28 days after infection. In our previous screening [10], infection involved up to 3–6×10^7^ Ba/F3 cells at the same density in multiple plates.

### Identification of integrated cDNA

To determine integrated cDNA sequences from IL3-independent Ba/F3 clones, genomic DNA was purified using the QIAamp DNA Blood Mini Kit (Qiagen) and used as template for a step-down PCR with primers flanking the inserted cDNA sequences:

att-f: 5′-TCCTCCCTTTATCCAGCCCTCACTCCTTCTCTAGG-3′

att-r: 5′-CTAAAGCGCATGCTCCAGACTGCCTTGGGAAAAGC-3′

PCR was conducted with KOD-FX DNA polymerase (Toyobo) using the following PCR conditions: 94°C for 4 min, 5 cycles of 98°C for 10 sec and 74°C for 8 min, 5 cycles of 98°C for 10 sec and 72°C for 8 min, 5 cycles of 98°C for 10 sec and 70°C for 8 min, 15 cycles of 98°C for 10 sec and 68°C for 8 min, and 68°C for 10 min. For the isolation of individual integrants, 25 µL of PCR product was separated by agarose gel electrophoresis and bands were purified from the gel using the QIAquick Gel Extraction Kit (Qiagen) and sequenced from the 5′ end by the Sanger method (Dana-Farber Cancer Institute Molecular Biology Core Facility).

For deep sequencing, PCR products amplified from independent Ba/F3 transformants within the same cDNA library were mixed at equimolar amounts (1–10 µL) and purified using the QIAquick PCR Purification Kit (Qiagen).

### Establishment of erlotinib resistant PC-9 cell line and inhibitor assays

PC9 cells were initially treated with 1 nM erlotinib and the concentration was increased to 3 nM, 10 nM, 100 nM and 1 µM as clones emerged over the course of approximately 2 months. To confirm resistance to erlotinib among the surviving PC9-ER cells, a dose response study was performed using the CellTiter-Glo® Luminescent Cell Viability Assay (Promega) and read by the 2104 EnVision® Multilabel Reader (PerkinElmer) after 48 hour treatment. Dose response to lapatinib was also quantified using CellTiter-Glo® after 48 hour treatment, as above.

### SOLiD library construction and sequencing

A sequencing library was generated from PCR products which were amplified from transfected DNA from each of 21 specimens. An average of approximately 10 IL3-independent BaF3 clones were derived from each screened library. We estimated that cDNA inserts would average 2 kb and that each BaF3 clone contains an average of 7 inserts, for a total of approximately 3×10^6^ bases of cDNA sequence PCR amplified from all 21 specimens. Sequencing of 10^10^ bases would thus generate greater than 3000-fold mean coverage which may be necessary to confidently recover larger and/or repetitive cDNA with lower PCR amplification efficiency.

The method used to generate the libraries is described in the Applied Biosystems SOLiD 4 Library Preparation guide. Briefly, for each library, the PCR products were concatatenated by ligation and then sheared. An adaptor was ligated to each end of each sheared fragment. One of the two adaptors contained a barcode sequence, which was unique for each library. Each library was amplified by 6–8 cycles of PCR. After quantification, the libraries were mixed in equimolar amounts and then subjected to emulsion PCR. The beads resulting from the emulsion were sequenced from each end - 75 bases from one end and 35 bases from the other end, on 4 lanes using an Applied Biosystems 5500 sequencer. The libraries were binned according to barcode sequence.

### Mapping and counting

Sequence data were mapped to the human reference genome, hg18, using the Genomic Resequencing and Whole Transcriptome Mapping modules contained in Lifescope 2.5 (Applied Biosystems.) The Lifescope 2.5 Exon Counts module was used in conjunction with a custom Python script to generate gene-level data, indentifying those transcripts which were present at a high level in each sample.

### Gateway Junction Detection

To identify the precise genomic coordinates of integrated cDNA, a pipeline for the detection of gateway fusion sequences was developed. Individual reads containing a piece of the Gateway attB sequence (5′-TTGTACAAAAAAGTTGG′-3′) were pulled from the Whole Transcriptome mapped data. Reads were selected if their color-space sequence matched the color-space sequence of the gateway, either forward, (5′-T0113110000021010-3′) or in reverse (5′-G0101200000113110-3′). A python script was developed to identify and report the precise coordinate of the gateway fusion for each selected read, taking into account forward vs. reverse strandedness, clipped mappings, and mappings crossing exon junctions. These gateway fusion coordinates were collected and reported at the gene level for each of the 21 multiplexed samples.

### Variant Analyses

Single Nucleotide Variants were called using DiBayes (Applied Biosystems) and annotated with information including exonic context (using RefSeq), known polymorphisms (using DBSNP 130), amino acid consequence, and frequency and quality of reads supporting each variant. Fusions genes were detected using the output of Lifescope's Splice Finder module and annotated against RefSeq using a Python script.

## Supporting Information

Figure S1 **Full length-att method for library construction.** **A.** cDNA was generated from PC9 and K562 cells using the In-Fusion SMARTer Kit and using the full length-att method, which modifies the former approach using the attB1_forward and attB2_reverse primers for first strand synthesis. cDNA was separated by agarose gel electrophoresis. λ indicates size ladder. **B.** Size fractionation of cDNA from PC9 and K562 cells generated using the full length-att method. **C.** BsrG1 digest of pDONR222 plasmids harboring cDNA from the K562 library generated using the full length-att method. **D.** Comparison between methodologies. Negative and positive controls are from the CloneMiner II kit (Invitrogen). The PC9ER library is described in Figure 3. CFUs indicates colony forming units.
(EPS)

## References

[1] Salmena L, Poliseno L, Tay Y, Kats L, Pandolfi PP (2011) A ceRNA hypothesis: the Rosetta Stone of a hidden RNA language? Cell 146: 353–358. 21802130 10.1016/j.cell.2011.07.014 PMC3235919

[2] Garraway LA, Janne PA (2012) Circumventing cancer drug resistance in the era of personalized medicine. Cancer Discov 2: 214–226. 22585993 10.1158/2159-8290.CD-12-0012

[3] Okayama H, Berg P (1982) High-efficiency cloning of full-length cDNA. Mol Cell Biol 2: 161–170. 6287227 10.1128/mcb.2.2.161-170.1982 PMC369769

[4] Gubler U, Hoffman BJ (1983) A simple and very efficient method for generating cDNA libraries. Gene 25: 263–269. 6198242 10.1016/0378-1119(83)90230-5

[5] Lee F, Yokota T, Otsuka T, Gemmell L, Larson N, et al (1985) Isolation of cDNA for a human granulocyte-macrophage colony-stimulating factor by functional expression in mammalian cells. Proc Natl Acad Sci U S A 82: 4360–4364. 3925454 10.1073/pnas.82.13.4360 PMC390413

[6] Kitamura T, Onishi M, Kinoshita S, Shibuya A, Miyajima A, et al (1995) Efficient screening of retroviral cDNA expression libraries. Proc Natl Acad Sci U S A 92: 9146–9150. 7568090 10.1073/pnas.92.20.9146 PMC40941

[7] Soda M, Choi YL, Enomoto M, Takada S, Yamashita Y, et al (2007) Identification of the transforming EML4-ALK fusion gene in non-small-cell lung cancer. Nature 448: 561–566. 17625570 10.1038/nature05945

[8] Harvey RC, Mullighan CG, Chen IM, Wharton W, Mikhail FM, et al (2010) Rearrangement of CRLF2 is associated with mutation of JAK kinases, alteration of IKZF1, Hispanic/Latino ethnicity, and a poor outcome in pediatric B-progenitor acute lymphoblastic leukemia. Blood 115: 5312–5321. 20139093 10.1182/blood-2009-09-245944 PMC2902132

[9] Hertzberg L, Vendramini E, Ganmore I, Cazzaniga G, Schmitz M, et al (2010) Down syndrome acute lymphoblastic leukemia, a highly heterogeneous disease in which aberrant expression of CRLF2 is associated with mutated JAK2: a report from the International BFM Study Group. Blood 115: 1006–1017. 19965641 10.1182/blood-2009-08-235408

[10] Yoda A, Yoda Y, Chiaretti S, Bar-Natan M, Mani K, et al (2010) Functional screening identifies CRLF2 in precursor B-cell acute lymphoblastic leukemia. Proc Natl Acad Sci U S A 107: 252–257. 20018760 10.1073/pnas.0911726107 PMC2806782

[11] Colleoni GW, Bridge JA, Garicochea B, Liu J, Filippa DA, et al (2000) ATIC-ALK: A novel variant ALK gene fusion in anaplastic large cell lymphoma resulting from the recurrent cryptic chromosomal inversion, inv(2)(p23q35). Am J Pathol 156: 781–789. 10702393 10.1016/S0002-9440(10)64945-0 PMC1876849

[12] Rho JK, Choi YJ, Lee JK, Ryoo BY, Na II, et al (2009) The role of MET activation in determining the sensitivity to epidermal growth factor receptor tyrosine kinase inhibitors. Mol Cancer Res 7: 1736–1743. 19808904 10.1158/1541-7786.MCR-08-0504

[13] Zhou W, Ercan D, Chen L, Yun CH, Li D, et al (2009) Novel mutant-selective EGFR kinase inhibitors against EGFR T790M. Nature 462: 1070–1074. 20033049 10.1038/nature08622 PMC2879581

[14] Mitra D, Brumlik MJ, Okamgba SU, Zhu Y, Duplessis TT, et al (2009) An oncogenic isoform of HER2 associated with locally disseminated breast cancer and trastuzumab resistance. Mol Cancer Ther 8: 2152–2162. 19671734 10.1158/1535-7163.MCT-09-0295

[15] Koivunen JP, Mermel C, Zejnullahu K, Murphy C, Lifshits E, et al (2008) EML4-ALK fusion gene and efficacy of an ALK kinase inhibitor in lung cancer. Clin Cancer Res 14: 4275–4283. 18594010 10.1158/1078-0432.CCR-08-0168 PMC3025451

[16] Houghton AN, Real FX, Davis LJ, Cordon-Cardo C, Old LJ (1987) Phenotypic heterogeneity of melanoma. Relation to the differentiation program of melanoma cells. J Exp Med 165: 812–829. 3102678 10.1084/jem.165.3.812 PMC2188299

[17] Kerbel RS, Man MS (1984) Single-step selection of unique human melanoma variants displaying unusually aggressive metastatic behavior in nude athymic mice. Invasion Metastasis 4 Suppl 1: 31–43. 6533097

[18] Rodig SJ, Mino-Kenudson M, Dacic S, Yeap BY, Shaw A, et al (2009) Unique clinicopathologic features characterize ALK-rearranged lung adenocarcinoma in the western population. Clin Cancer Res 15: 5216–5223. 19671850 10.1158/1078-0432.CCR-09-0802 PMC2865649

